# The Role of Automated External Defibrillator Use in the Out-of-Hospital Cardiac Arrest Survival Rate and Outcome: A Systematic Review

**DOI:** 10.7759/cureus.47721

**Published:** 2023-10-26

**Authors:** Mohamed O Elhussain, Fatima k Ahmed, Nafisa M Mustafa, Doaa O Mohammed, Ibrahim M Mahgoub, Namarig A Alnaeim, Ragda Ali, Noura Bushra, Hassan K Ahamed, Nadir Abdelrahman

**Affiliations:** 1 Family Medicine-Geriatrics, Michigan State University, East Lansing, USA; 2 Internal Medicine, Sudan Medical Specialization Board, Khartoum, SDN; 3 Family Medicine-Geriatrics, Human Medicine, Michigan State University, East Lansing, USA

**Keywords:** ventricular fibrillation, out patient cardiac arrest, out of hospital cardiac arrest, automated external defibrillator, public access defibrillation, pad, cpr, ems, ohca, aed

## Abstract

Out-of-hospital cardiac arrest (OHCA) remains a significant cause of death. The chance of survival significantly increases when immediate defibrillation with an on-site automated external defibrillator (AED) is available. Our aim is to systematically evaluate the impact of public access defibrillators (PAD) on the outcomes of outpatient cardiac arrest. We conducted a systematic review of the data from global studies on the role of bystander and emergency medical service (EMS) interventions, primarily focusing on the usage of AEDs, during OHCA events. The results highlight the critical significance of PADs in improving survival outcomes in OHCA settings. The majority of OHCA incidents occurred in private residences, but public spaces such as schools and airports had better outcomes, likely due to AED accessibility and trained individuals. Placing AEDs in public areas, especially high-risk zones, can boost survival chances. Timely defibrillation, particularly by bystanders, correlated with better survival and neurological conditions. The review emphasizes the importance of widespread cardiopulmonary resuscitation (CPR) and AED training, strategic AED placement, and continuous monitoring of interventions and outcomes to enhance survival rates and neurological recovery after OHCAs. This systematic review showed that bystander interventions, including CPR and AED usage, significantly increased the survival rate. Overall, immediate response and accessibility to AEDs in public areas can significantly improve outcomes in OHCA events.

## Introduction and background

Every year, millions worldwide are affected by cardiac arrest, a life-threatening event that occurs suddenly and unexpectedly. Improving survival chances is crucial for rapid intervention within the critical minutes after cardiac arrest. Defibrillation, which involves delivering an electric shock to the heart, is a well-established and successful technique for restoring a normal heart rhythm, which ultimately reduces the negative health effects and death rates associated with sudden cardiac arrest. Public access defibrillators (PADs) refer to the use of automated external defibrillators (AEDs) to treat out-of-hospital cardiac arrests (OHCA) outside the emergency hospital settings or by individuals who are not part of the conventional emergency medical services. The availability and prompt utilization of defibrillators outside hospitals have been a persistent challenge. OHCA remains a significant cause of death, with over 350,000 cases occurring annually in the US, resulting in a survival rate of only 8-10% [1]. The chance of survival significantly increases to 50%-74% when immediate defibrillation with an on-site AED is available [2,3-4].

In recent years, there has been increasing recognition of the importance of public access to defibrillators in improving outcomes for OHCA. Nevertheless, evaluating the overall effect of having widespread access to AEDs on these essential outcomes, such as morbidity, mortality, and survival, continues to be a central focus within the emergency medicine discipline. In this study, we evaluate the impact of public access defibrillators on the outcome of OHCA.

## Review

Methodology

Eligibility Criteria

This systematic review was structured with reference to the Preferred Reporting Items for Systematic Reviews and Meta-Analyses (PRISMA) guidelines for reporting, and we adhered to its principles. Most parts of the review were conducted through an online tool known as Covidence (Veritas Health Innovation Ltd., Melbourne, Australia), and all databases were imported from PubMed.

Studies included in the review meet the following criteria: studies involving individuals of all age groups who experienced OHCA from the year 2000 to the present and studies conducted in all countries with OHCA and the availability of PADs. On the other hand, all studies reporting on inpatient cardiac arrest cases or were conducted before the year 2000 were excluded from this systematic review.

Selection Process

The screening and selection process for this systematic review was cautiously conducted using the Covidence platform, a widely utilized tool for systematic review guidance. Two independent reviewers initially screened the first set of titles and abstracts of studies. In cases of conflicts or disagreements, a consensus was reached through discussion, and issues were resolved. Subsequently, the same two reviewers independently conducted full-text screening for eligible studies, using the screening tools provided by Covidence. This meticulous and standardized process ensured that only relevant studies meeting the predefined inclusion and exclusion criteria were included in the systematic review, enhancing the reliability and accuracy of the study selection process. The search strategy included keywords and MeSH terms related to out-of-hospital cardiac arrest, public facilities and access, and defibrillator use and related keywords. In this systematic review, we conducted a rigorous screening process to identify relevant studies for our analysis. Initially, a total of 345 references were imported for screening, all of which were considered potential studies. We began by screening these studies against their titles and abstracts, resulting in the exclusion of 188 studies. Following this initial screening, we assessed the eligibility of 157 studies by reviewing their full texts. At the end of this comprehensive screening process, we identified and included 30 studies that met our predefined criteria for inclusion in our analysis.

Data Items

The systematic review collected a range of data items to thoroughly assess the outcomes of interest. For the outcome, which focused on the comparison between bystanders' use of AEDs and EMS, data items included survival rates and neurological outcomes.

Data Collection and Quality Assessment

Data collection and quality assessment for this systematic review were carried out with a standardized approach. Using the online tool Covidence, two independent reviewers were responsible for data extraction, and conflicts were resolved consistently. Simultaneously, another pair of reviewers independently conducted quality assessments for the extracted data using the strengthening the reporting of observational studies in epidemiology (STROBE) framework. To ensure adherence to best practices, templates following the PRISMA guidelines were utilized for both data extraction and quality assessment.

Risk of Bias Assessment and Reporting of Bias

The risk of bias assessment for each included study was conducted following the STROBE framework. This assessment tool ensured a comprehensive evaluation of potential biases in each study. Two reviewers were responsible for assessing the risk of bias in each study, working independently to maintain objectivity and focus.

Synthesis Method

In this systematic review, we employed the narrative synthesis method as the primary approach for synthesizing the evidence. To facilitate the presentation and synthesis of the data, we utilized Microsoft Excel as a valuable tool. The data were meticulously organized and displayed in a table and bar charts. This approach was chosen to enhance the comprehensibility and accessibility of the study results, allowing for a clear and concise representation of the findings. This methodological choice was made to ensure that readers and stakeholders can easily grasp and interpret the synthesized evidence for informed decision-making. We utilized quality control templates to assess the methodological quality and risk of bias in the included studies. Specifically, we employed the PRISMA and STROBE checklists. These validated tools provided comprehensive guidelines to ensure a thorough and standardized assessment across all studies. As per PRISMA recommendations, we assessed the overall certainty of the evidence using the STROBE framework.

Results

**Figure 1:**
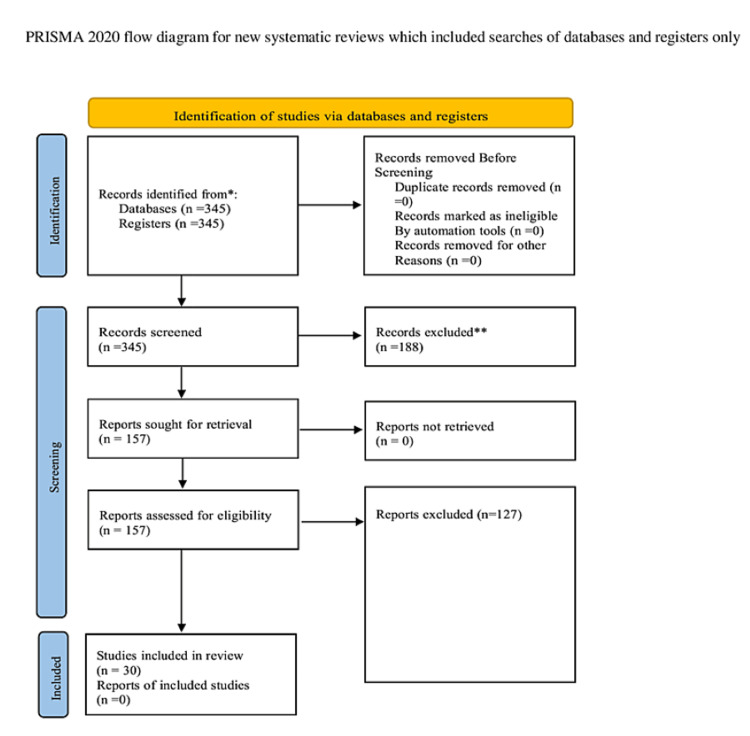
Prisma flow chart

We identified 345 articles using various search methods, ensuring there were no repetitions. After assessing their relevance to our current research through their titles and abstract contents, we discarded 188 articles due to their lack of pertinence. This left us with 157 articles. Upon checking for full-text availability, an additional 127 articles were eliminated. Of the articles left, 30 met our eligibility standards and were chosen after a thorough review. The detailed PRISMA flow diagram is presented in Figure 1 below.

A summarized table of these studies can be viewed in Table 1 below, which presents the results from various studies, each employing different research designs. These studies have specifically examined the key outcomes based on interventions made by bystanders or the emergency medical service (EMS). The table consolidates the study IDs and represents the comparative impact of each intervention on the outcome.

**Table 1:** Total studies included N: Neurological outcome; S: Survival outcome; EMS: Emergency medical service; NA: Not available

Study ID	Country	Total Number of Participants	Bystander Survival (S) or Neurological Outcome (N)	EMS Survival (S) or Neurological Outcome (N)
Myerburg et al. 2002 [5]	United States	420	17% S	9% S
Nakahara et al. 2015 [6]	Japan	167912	40.70% S	15% S
Mitani et al. 2014 [7]	Japan	NA	69% N	35% N
Kiyohara et al. 2017 [8]	Japan	NA	77% S	35% S
Sun et al. 2020 [9]	Denmark	653	32.30% S	NA
Myat et al. 2019 [10]	Australia	1299784	37.7% N	22.6% N
Nakashima et al. 2019 [11]	Japan	28019	44% S	31.80% S
Kishimori et al. 2020 [12]	Japan	1743	29.8% N	9.7% N
Siddiq et al. 2013 [13]	Canada	1310	12.20% S	NA
Caffrey et al. 2002 [14]	United States	354	13% S	7% S
Culley et al. 2004 [15]	United States	NA	50% S	NA
Haskins et al. 2020 [16]	Australia	NA	55.50% S	28.80% S
Kiguchi et al. 2019 [17]	Japan	NA	51.80% S	25.50% S
Kitamura et al. 2010 [18]	Japan	312,319	86% S	78% S
Kiyohara et al. 2019 [19]	Japan	409	52.20% S	NA
Swor et al. 2013 [20]	United States	47	15.10% S	NA
Ringh et al. 2015 [21]	Sweden	6532	70% S	31% S
Murakami et al. 2014 [22]	Japan	6190	10% S	NA
Agerskov et al. 2015 [23]	Denmark	2080	71.40% S	NA
Kiyohara et al. 2016 [24]	Japan	9978	19.4%N	3% N
Nielsen et al. 2013 [25]	Denmark	NA	69% S	NA
Capucci et al. 2016 [26]	Italy	3271	41.40% S	5.90% S
Torney et al. 2020 [27]	UK	NA	30.10% S	16% S
Iwami 2012 [28]	Japan	NA	38.50% S	18.20% S
Sun et al. 2019 [29]	Denmark	673	31.30% S	NA
Kitamura et al. 2016 [30]	Japan	NA	38.5% N	18.2% N
Haskins et al. 2022 [31]	Australia	NA	39.60% S	24.20% S
Marenco et al. 2001 [32]	Canada	2172	40.10% S	NA
Shibahashi et al. 2021 [33]	Japan	562	61.20% S	NA
Odom et al. 2022 [34]	United States	NA	19.70% S	13.80% S

**Figure 2:**
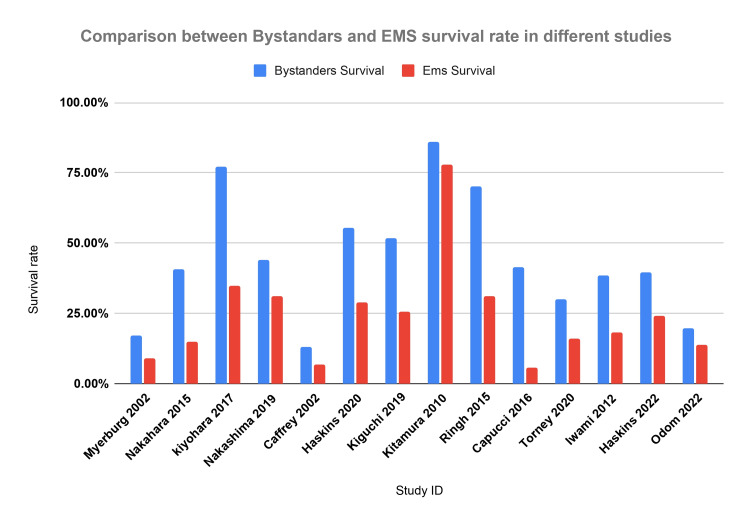
Comparison between bystander and EMS survival The data have been represented as percentages (%). EMS: Emergency medical service Myerburg et al. 2002 [5], Nakahara et al. 2015 [6], Kiyohara et al. 2017 [8], Nakashima et al. 2019 [11], Caffrey et al. 2002 [14], Haskins et al. 2020 [16], Kiguchi et al. 2019 [17], Kitamura et al. 2010 [18], Ringh et al. 2015 [21], Capucci et al. 2016 [26], Tourney et al. 2020 [27], Iwami 2012 [28], Haskins et al. 2022 [31], Odom et al. 2022 [34]

In Figure 2, we present a comparative analysis of survival rates between bystander-initiated and EMS-initiated interventions across various studies, identified by their respective IDs. Notably, the figure emphasizes the significant increase in survival rates when interventions are carried out by bystanders compared to those by EMS.

**Figure 3:**
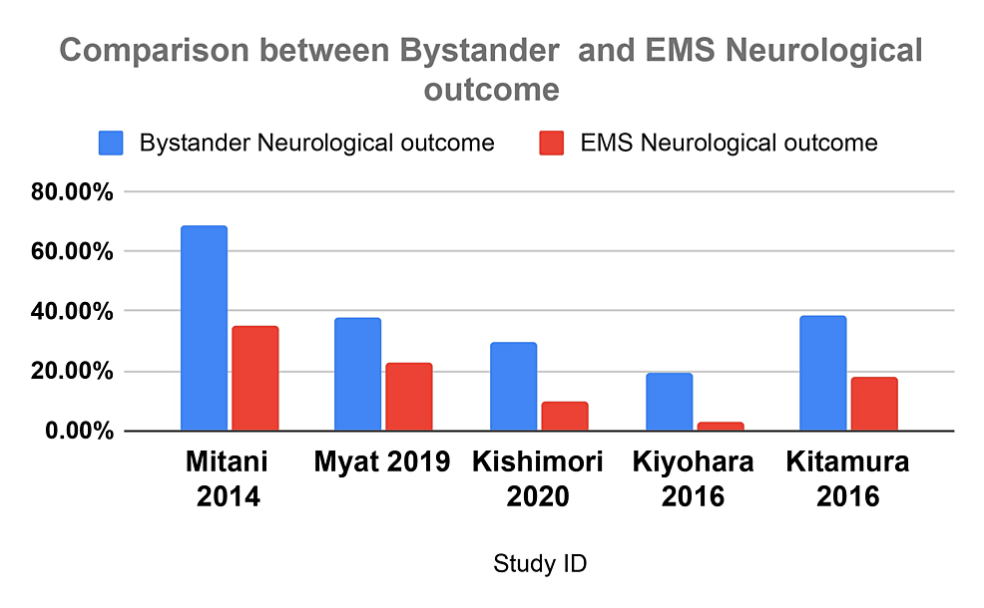
Comparison of neurological outcome between bystander and EMS The data have been represented as percentages (%). EMS: Emergency medical service Mitani et al. 2014 [7], Myat et al. 2019 [10], Kishimori et al. 2020 [12], Kitamura et al. 2016 [18], Kiyohara et al. 2016 [19]

Figure 3 illustrates the comparison of neurological outcomes between interventions by bystanders and EMS across various studies.

Discussion

This review synthesizes data from various global studies on the role of bystander and EMS interventions, primarily focusing on AED usage, during OHCA events. The results of this systematic review, following PRISMA guidelines, underscore the critical significance of PAD in improving survival outcomes in OHCA settings. Our objective is to evaluate the impact of PADs on the outcome of OHCA. The present investigation provides significant insights into the effectiveness and challenges associated with the deployment and use of PADs in various settings.

Epidemiology and Outcomes

The global statistics suggest that a substantial proportion of cardiac arrests occur at home as 59% of OHCAs occur in private residences [35]. However, the odds ratio of survival of 1.75 suggests a significantly higher chance of survival when an AED is used in public areas, compared to instances where it is not. The statistics from Japan further emphasize the lifesaving potential of PADs, with increasing PAD use being directly associated with better neurologic outcomes following ventricular fibrillation OHCAs [36].

Location and Its Influence on the Outcomes

OHCA locations play a crucial role in outcomes. A retrospective study conducted by Frank et al. on the locations of nonresidential OHCAs in the city of Pittsburgh over a three-year period: implications for automated external defibrillator placement, aimed to identify the sites of OHCAs that occurred in nonresidential areas of Pittsburgh, and to ascertain if there are specific "high-risk" locations where the installation of AEDs could be beneficial [35]. The study showed that, between January 1, 1997, and December 31, 1999, the city of Pittsburgh experienced 971 OHCAs. Out of these, 575 or 59% took place in private homes, while 396 or 41% happened in nonresidential settings [35]. This prevalence in private settings highlights the potential need for increased public awareness and training in these areas, along with easier access to AEDs. The review emphasizes the pronounced benefits of immediate defibrillation using AEDs further. Timely defibrillation, especially by bystanders, frequently correlated with better survival and neurological conditions.

Bystander vs. EMS Intervention

Most research emphasizes the survival benefits of early interventions by bystanders, for example, the study done between 2008 and 2015, by Kiyohara et al. It showed that the use of publicly accessible AEDs together with bystander-administered CPR in schools boosted survival rates by roughly four times for school children indicating faster defibrillation by bystanders than by emergency responders [37]. By looking at the data provided, it is evident that the combination of bystander cardiopulmonary resuscitation (CPR) with the use of AED significantly increased the odds ratio of survival (1.75, 95% CI 1.23-2.5, p < 0.002). This result suggests that bystanders equipped with both CPR training and AEDs can significantly impact patient outcomes positively [38]. Even when bystanders did not provide CPR but used an AED, the survival rate was still at 17% compared to 9% where no intervention was applied [5,9]. This highlights the importance of AEDs in resuscitation efforts initiated by the bystander that had a significant impact on the survival rate and the neurological outcome of the OHCAs in the pediatric age category [17].

Survival Rates in Different Settings

A key observation was that survival rates were significantly higher in public locations, standing at 51.8%, compared to residential settings, which were at 22.5%. Furthermore, neurological outcomes, an essential measure of post-resuscitation quality of life, were 25.5% in public settings and 18.6% in residences [39]. A study in Japan sought to assess the impact of using PADs and CPR initiated by bystanders on the survival of pediatric patients who experienced OHCA on school grounds. Patients with non-traumatic OHCA from elementary, junior high, and high school/technical colleges were included in the study conducted between April 2008 and December 2015. There was a substantial improvement in the 30-day survival rate with a positive neurological outcome, increasing from 38.1% in 2005 to 56.5% in 2015 (P-value for trend = 0.026) [39].

30-Day Survival Rate

Over the recent years, the recognition of bystanders' crucial role in enhancing survival rates for OHCAs has increased. Consequently, there has been a notable rise in the use of PAD prior to the arrival of EMS. A study in Denmark evaluated the 30-day survival rate, aiming to identify the percentage of OHCA cases where an AED was used before the ambulance's arrival. Additionally, we examined the percentage of OHCA situations with an accessible AED located within 100 m. In 20 cases (3.8%, 95% CI 2.4-5.9), an AED was used, considering its availability within a 100 m radius. However, at the time of the OHCA, only 15.1% of the cases had an AED both within 100 m and available for use [23]. For OHCAs with an initially shockable rhythm, the 30-day survival rate was 64% when an AED was used before the ambulance's arrival and 47% when no AED was used. This finding underscores the importance of immediate on-site interventions using AEDs [23].

Neurological Outcome

The life-saving potential of rapid intervention during cardiac emergencies is crucial, and the PAD has played a critical role, particularly outside of hospital settings. Recent research has highlighted its efficacy, especially among school-aged children who experience ventricular fibrillation (VF); the study has indicated that those children who were administered a shock by a bystander using a PAD displayed a notably better neurological outcome compared to those who waited for EMS intervention [39]. Recent research from Japan has shed light on the advantages of using AED in public areas. This study found that using AEDs in public settings resulted in more favorable neurological outcomes following OHCAs caused by VF than when used in residential locations [39]. This underscores the importance and potential of widespread PAD availability and bystander intervention.

Implications for Practice and Policy

The benefits of early CPR and defibrillation emphasize the importance of widespread training in CPR and AEDs. Placing AEDs prominently in public areas, especially in high-risk zones such as train stations, can boost survival chances. Regular tracking of OHCAs, especially in densely populated areas, will help in the strategic placement of AEDs and specialized training initiatives. While the current data are robust, further research studies on subgroups, such as children, can fine-tune recommendations (morbidity and mortality).

Limitations

While offering a broad view of OHCA interventions and outcomes, this review is not without limitations. Variability in data collection methods and study demographics may lead to inconsistent conclusions. Some studies found no clear link between AED use and sustained return of spontaneous circulation. The benefits of PAD were less evident in unwitnessed OHCAs or non-cardiac cases. Although OHCAs in educational settings showed better outcomes due to bystander aid, adjusted survival rates were similar to public places. Other results, such as unchanged 30-day survival for non-EMS witnessed OHCAs or inconsistent AED benefits, highlight potential confounders. A comprehensive approach combining PADs, CPR training, and swift EMS response is necessary.

Future research should focus on specific subgroups, training impacts, and factors influencing AED availability.

## Conclusions

The systematic review highlights the importance of immediate response, particularly from bystanders, in improving both the survival rate and the outcomes of OHCAs. The findings emphasize the significance of raising awareness, providing training, and ensuring easy accessibility to AEDs in public areas. These interventions have the potential to improve the chances of survival and overall results of OHCAs. Therefore, it is essential to focus on the widespread use of CPR and AED training, strategically placing AEDs in public spaces, and continuously monitoring interventions and outcomes to enhance survival rates and neurological recovery after OHCAs.
